# The Hospital. Nursing Section

**Published:** 1903-10-24

**Authors:** 


### The Hospital.

### Nursing Section.

Contributions for this Section of "The Hospital" should be addressed to the Editor, "The Hospital" Nursing Section, 28 & 29 Southampton Street, Strand, London, W.C.

No 891.—Vol. XXXV. *SATURDAY, OCTOBER* 24, 1903.

### Notes on News from the Nursing World.

### WORKHOUSE NURSING AND THE PROPOSED "QUALIFIED NURSE."

On Thursday afternoon, next week, at half-past four, a discussion on the report of the Departmental Committee on Nursing will take place at the Governors' Hall, St. Thomas's Hospital, under the auspices of The Hospitals Association. It will be opened by Miss Gibson, matron of Birmingham Infirmary, and Dr. Savill, formerly medical superintendent of Paddington Infirmary. Tickets of admission may be had on application to Mr. Sydney Phillips, Hon. Sec. of The Hospitals Association, The Hospital Building, 29 Southampton Street, Strand, W.C.

### VOLUNTEER NURSES FOR THE BALKANS.

The result of the statement in our columns that nurses are needed in European Turkey is that several experienced nurses have written to us offering their services, one of them having been matron and another a sister in well-known hospitals. But, though these offers justify our remark that there are English trained nurses ready to go to the Balkans, we are unable to afford our correspondents any information as to an organisation under whose auspices they might venture into the districts where they are wanted. Such an organisation does not, in fact, appear to exist; and so long as this is the case, it is impossible for us to give any advice except the warning to nurses not to think of going on their own account.

### THE FAILURE OF A NURSING HOME.

Last month we warned our readers against the risks of starting a nursing home with inadequate capital. We did not on that occasion emphasise the equally essential need of business capacity on the part of anyone embarking in such an enterprise. An exceedingly sad illustration of the folly of a person deficient in this indispensable qualification has just come under our notice. For the last two or three years a large, well-ordered, and apparently flourishing nursing home has existed in one of our sea-side resorts in the West of England. The house stands in its own grounds, is admirably furnished and fitted up for the purpose required, and had a domestic staff of seven, including a gardener and an odd man. The nurses, nearly a score in number, were well cared for in every way, the home has been held in high estimation by the doctors and residents in the neighbourhood, and the scale of fees paid by patients has been of a character that should be remunerative. Unfortunately, however, the owner had not mastered the vital lesson of cutting her coat according to her cloth. Although she put the whole of her available capital—a considerable sum—into the enterprise, she failed to live within her means, and has now been overtaken by the inevitable result. In other words, her creditors have come upon her, and the home will either have to be given up or pass into other hands. But what else could be expected from a lady who admits that she never kept accounts?

### ELECTION OF THREE MATRONS.

At a meeting of the Metropolitan Asylums Board on Saturday Miss Emily Baker was elected matron of the High Wood Ophthalmia Schools at a salary of £100 a year, rising by annual increments of £5 to £150, with furnished apartments; Miss Elizabeth Palmer, matron of St. Anne's Home, Herne Bay, at a salary of £75 a year, rising to £100, with the usual emoluments; and Miss Grace Kingcott, matron of the Millfield Seaside Home, Rustington, at a salary of £75, rising to £90, with the usual emoluments. The there ladies were previously interviewed by the Board. These appointments gave rise to considerable discussion, in the course of which charges of favouritism were brought forward by members opposed to the committee's recommendation, a charge which was indignantly denied. It was retorted that the cause of the opposition to the nurses invited to come before the Board was due to a certain amount of jealousy on the part of members who had their own particular candidates. One member stated that in answer to the advertisements for these posts they had received altogether no fewer than 308 applications, yet two out of the three ladies appointed that morning were from their own staff. He agreed that it was right to encourage their own staff to look forward to promotion, but he thought that the outside applicants had been put to a great deal of unnecessary trouble in replying to the advertisements.

### THE AGE QUESTION.

The suggestion of the author of an article in our issue of October 10 that elderly nurses might find an opening to act as foster-mother to children who are under the care of Poor Law guardians, has provoked a bitter cry from a correspondent who seems to assume that women over 40 are no longer regarded as eligible nurses. This is not the case by any means, and we hope that it will never be so. There may be cases in which nurses exceeding 40, and seeking a new post, find a difficulty in obtaining one because of their age. But as "A Matron" recognises in her letter to us to-day, the difficulty is not confined to nurses, nor even to one sex. Women and men in all departments of life often find it hard to obtain employment after they have passed the age of 40. The question of age should, of course, be practically immaterial, or at least quite subsidiary to that of capacity. Indeed, we believe it to be so, as a rule, where persons of any age are doing good service in posts they have held for years. There are many nurses considerably over 40 in hospitals, infirmaries, and engaged in private work, and some of these are still the best of the profession, because the most experienced. We do not deny that there may be now and again instances of capable nurses of middle age being told that they are too old for vacancies, but they suffer in common with governesses, clerks, and all sorts of workers, not because they are members of a profession in which the arbitrary enforcement of an age limit would be distinctly injurious to the interests of the community.

### A NURSE FOR FORTY-FOUR YEARS.

On Friday last there passed away at Morecambe, in the person of Mrs. Mary Milnes, a remarkable woman. Mrs. Milnes, who was seventy-two at the time of her death, retired a year ago from the post of superintendent nurse at the North Bierley Union Infirmary, after having acted as nurse for forty-four years. The first fourteen years were spent at the West Riding Asylum, Wakefield, and the remaining thirty at the North Bierley Union Workouse, Bradford. Until she was seventy-one, she carried on her work as superintendent nurse to the satisfaction of the Guardians and of the Local Government Board. She resigned on her own initiative, and it was not apparent to any one but herself that her health was giving way. At Wakefield Mrs. Milnes served under Mr. J. D. Cleaton, and Sir James Crichton Browne, and, both as a nurse and as a woman, she was greatly esteemed.

### NURSES AND THE REALITIES OF LIFE.

Writing on the controversy which has been raised in the leading journal on "Lady Doctors and General Hospitals" one of the surgeons to the Royal Free Hospital admits that a medical student, whether man or woman, has to face "the realities of life" and to touch much that is disagreeable. "But the same," he points out, "may be said of lady nurses, now universally employed at London hospitals," and he adds, "It is difficult to understand why scenes that are considered to be degrading for women who are medical students and doctors should be thought seemly and becoming for the similar class of young women who happen to be nurses." Quite so. Arguments which apply in one case would certainly apply in the other. But so far as nurses are concerned, we entirely dissent from the proposition that the scenes which they are compelled to witness are, in any sense, degrading to them as women. If the work of a nurse has not an elevating influence upon the woman who is engaged in it, she has mistaken her vocation.

### THE LEICESTER NURSES' HOME AT NORWICH.

At the quarterly meeting of the Governors of the Norfolk and Norwich Hospital reference was made to the opening of the Leicester Nurses' Home. As we stated in July, in connection with the opening of the home, the Earl of Leicester has not only defrayed the entire cost of the building, but has also endowed it with the sum of £5,000. In spite of this, fears seem to have been expressed by friends of the hospital that the expenses of maintaining the home might be a drag on its finances. It was, however, pointed out at the meeting that the home is large enough for the accommodation of a staff of private nurses, in addition to those employed in the hospital, and that their earnings will supplement the income derivable from the endowment. There really ought not, after Lord Leicester's munificent gift, to be any pecuniary difficulties in connection with the home, which, instead of being in any sense a tax on the finances of the hospital, should, by conducing to the efficiency of the nursing, increase the popularity of the institution.

### "A NEAT, STYLISH, AND ELEGANT MATRON."

An American physician, writing to the superintendent of nurses of a large training school in the United States, informs her that he wants "a graduate nurse to superintend and manage a small hospital of about 20 beds." He then proceeds to set forth his requirements as follows:—"She should have some executive ability. Age, 25 to 32; height 5 ft. 3 in. to 5 ft. 6 in.; weight, 118 to 145 lbs. Fine personal appearance, neat and stylish, elegant form, well developed, good looking, dignified, pretty mouth and teeth, splendid health, elegant disposition, popular, good education, medium complexion, pretty eyes, first-class references. Applicants will please send recent photographs, which will be returned if desired. State whether single, married, or widow, where born and raised, city or country, and salary expected."

### "SUPERNUMERARY ASSISTANT NURSES."

The matron of the Poplar and Stepney Sick Asylum, in her report to the Visiting Committee, having asked that the five supernumerary assistant nurses still employed in the asylum should be put on the permanent staff, the managers have decided to adopt the proposal, subject to the approval of the Local Government Board. So long as the work in the wards is heavy enough to render their services essential, we do not suppose that the Local Government Board will object. The five supernumeraries are really probationer nurses on their trial, and if satisfactory are put on the regular staff as vacancies occur. They will receive the same training as the probationers, and the time spent on the temporary staff will count in their nursing.

### STEWARDESSES WITH NURSING TRAINING.

We observe that an agency is advertising for "several ladies as stewardesses for ocean line of steamers who must have had nursing training." What is the meaning of this? The movement having in view the employment of fully-trained nurses on ocean-going steamers is an excellent one, but we have not heard that it has made any progress, nor have we learnt that there is any demand for nurse stewardesses, although there are many applicants for such posts.

### NURSING AT MEERUT.

At Meerut there are three military hospitals, but all the acute cases are sent to one of the two smaller, where there has hitherto been a lady superintendent and four sisters belonging to Queen Alexandra's Military Nursing Service for India. In future there are to be five sisters in addition to the lady superintendent. There are also about twenty orderlies of whom about half are trained. They are chosen from the regiments in the station and are trained by the medical officers and sisters. They have to pass an examination and receive a certificate. The sisters, as a rule, do not each as in England have a ward, but alternately take charge of the whole hospital for a morning, an afternoon, or at night. Night duty is generally taken a week at a time, but sometimes it is arranged for the sisters to have a day off before and after each night on duty. In the hot weather the orderlies have either four or six hours on duty with 24 hours off. There is also a native army hospital corps of four classes—ward servants, cooks, water-carriers, and sweepers. In the other two hospitals there are no sisters and as a rule no orderlies; the "nursing" is done by the ward servants.

### CAMBERWELL GUARDIANS AND THEIR MATRON.

In consequence of the opening of the new extension of Camberwell Workhouse Infirmary, there has been a great increase of work devolving upon the matron, Miss Marquardt. For instance, the Infirmary Committee reported at the last meeting of the Guardians that nearly 28,000 articles were cut out, made up, and marked, under her special direction. The Committee deeming it only right to express their appreciation of the able and zealous manner in which the matron has discharged her duties the Guardians unanimously decided to grant her a further leave of absence for two weeks. The Infirmary Committee also reported at the same meeting that at the recent Sick Cookery Examination, 13 nurses were examined, of whom eight secured first-class and four second-class marks, only one failing to satisfy the examiner, and she obtained 55 per cent. of the marks.

### TAVISTOCK COTTAGE HOSPITAL.

The managers of Tavistock Cottage Hospital have appointed Miss Kate Ray, of the Seamen's Hospital, Greenwich, as matron at a salary of £60 a year. There were over a hundred candidates for the post vacated by Miss Hosking, who is retiring into private life after 14 years' useful work. Ill-health is mainly the cause of her retirement, and that she discharged her duties satisfactorily may be gathered from the fact that the friends and supporters of the hospital, with some of the former patients, have subscribed the sum of £50 on her behalf.

### DISTRICT NURSING IN KILBURN.

The initial annual public meeting of the Kilburn and West Hampstead Nursing Association, which has just been held at St. Mary's Hall, Kilburn, showed the extreme need which had hitherto existed in the district for a Queen's nurse. The first nurse started work in September last, and was joined soon after by two more, but it was found that the poor greatly wanted the assistance of a maternity nurse, and the three Queen's nurses were debarred from this work. Accordingly, in February of the present year a maternity nurse from a training home was engaged to work quite independently for the Association, the services of one of the Queen's nurses being dispensed with. About the same time, owing to representations made by one of the local clergy, it was determined to add West Hampstead to the sphere of the nurses' duties. The expenses at the outset have been very heavy, and though most of the places of worship in the neighbourhood have contributed to the support of the Association there is an adverse balance of £4 14s. 9¼d. Two hundred and five cases were attended during the year, and 5,085 visits paid. That the work of the nurses is appreciated by the poor has been indicated by their grateful expressions, and by the fact that many thank-offerings of money have been sent. Further funds are asked for and also gifts of bed-linen, night-clothes, and old linen. These latter might be contributed by ladies in the district who cannot perhaps afford to help in money.

### MAKING TWO ENDS MEET AT CORWEN.

The admirable manner in which the Corwen District Nursing Association is managed has caused a local paper to commend the vigour and business like methods of the committee to the imitation of the people of Llangollen. A secret of the success of the Corwen Association, whose nurses attended 75 cases during the year just closed, paying upwards of 2,000 visits, and whose expenditure was more than covered by income, is that meetings of the committee are held every month. At the meetings the reports of a band of collectors are considered, and the most careful attention is given to all details. In these circumstances, we are not surprised that in addition to making two ends meet, the Corwen Association is steadily accumulating a useful reserve fund.

### MALDON NURSING ASSOCIATION.

At the annual meeting of the Maldon and Heybridge Nursing Association last week the report was submitted by the honorary secretary and adopted at the instance of the chairman. It is worthy of note that since the expenditure has increased the receipts have also so greatly improved that there is now a balance in hand of £34. The nurses last year paid nearly 5,000 visits and were on duty 33 nights. It was decided to contribute £10 out of the balance to the training home at Leytonstone, where certain probationers are trained for two years and others for a shorter period which was not specified.

### OUR CHRISTMAS DISTRIBUTION.

We have to acknowledge, with many thanks, the receipt of contributions to our Christmas Distribution of clothing from Miss Ada Medforth, St. Bernard's Kennels, Roos, Hull; Miss A. M. Grafton, Wessington Court, Woolhope, Herefordshire; and Miss Henslowe, Preston Vicarage, Weymouth.

### SHORT ITEMS.

The ss. *Carisbrook Castle*, which arrived at Southampton on Saturday, had on board Nursing Sister H. Rooke, who is returning from South Africa in consequence of the reduction of establishment.—The Kidderminster Guardians have complied with a request from the nurses at the workhouse infirmary that their day's holiday should commence when they go off duty on the evening previous to taking the holiday.—The Tonbridge Guardians at their last meeting gave testimonials to four of the nurses, who successfully passed their examinations under Dr. Bishop, the medical officer, and Miss Tisdell, the superintendent nurse.—A number of the night attendants at Wadsley Asylum have petitioned for one night off duty every ten days instead of every fourteen days as at present, and the petition has been referred by the West Riding General Asylums Committee to the Chairman and Medical Superintendent of each of the three pauper asylums for consideration and report.

### The Examination of the Urine.

By J. K. WATSON, M.D.

(Concluded from page 28.)

The Examination and Collection of the Urine (*continued*).

X. The nurse should always test for the presence of sugar as a routine practice, although the medical man may not always consider it necessary. We have seen the more important characters of diabetic urine. There are, as in the case of albumin, many tests for sugar and of these that known as Fehling's test is the most commonly used. This and similar tests depend on the fact that the glucose in the urine has the power when heated of taking away some of the oxygen from the copper solution which is used and, as we say, reducing it and thereby producing another compound of copper of a different colour. To ensure the Fehling's solution being fresh and active it is best to keep its ingredients in two bottles labelled "Fehling No. 1" and "Fehling No. 2," and to mix equal parts of these in the test-tube at the time we perform the test. This mixture, then, constitutes Fehling's solution, and its deep blue colour should persist after a little is boiled in a test-tube. After, then, boiling an inch of Fehling's solution (made by taking one half inch from bottles 1 and 2) for two minutes, we add a few drops of the suspected urine (which should be free from albumin) and again boil, a yellowish red precipitate forms if sugar is present in considerable amount. If there be no precipitate then add as much urine as there was Fehling and boil again and then set aside. If in a few moments the solution remains clear there is no sugar present. There are certain fallacies connected with this test since certain other substances than glucose have a reducing action on the copper, but it is not in the scope of these remarks to consider them. The nurse should make use of one or more of the following tests for sugar if she has got a reaction with Fehling's test, in order to confirm her result. Pavy's solution, which acts in the same way as does Fehling's solution may be used instead.

Trommer's test is performed as follows:—Take about 2 inches of urine in a test tube and add about half the amount of liquor potassæ and then drop in carefully a solution of sulphate of copper, drop by drop. A precipitate forms, if sugar is present, which re-dissolves. Now boil the mixture a thick precipitate occurs, first yellow, then orange, then reddish brown in colour.

Picric acid is used to detect sugar as well as albumin. Boil an inch of picric acid and liquor potassæ in a test tube. Then add the urine (about 1 inch) and continue the boiling. If sugar is present the solution becomes of a dark red colour depending for its depth on the amount of glucose present. Normal urine, especially if high coloured, may cause a darkening of the fluid but the colour is not nearly so dark as that due to sugar. Test the picric acid, to see if it is pure, before using it, as it may darken of itself when heated with liquor potassæ if it is impure.

Böttger's or Nylander's test is sometimes used and is considered a delicate one. Heat the urine with a little carbonate of soda and a little subnitrate of bismuth. If sugar is present the solution becomes black owing to the power of glucose to reduce the bismuth compound when in an alkaline solution to a black powder (oxide of bismuth).

Moore's test for sugar is not recommended, as it is not reliable for detecting small amounts of sugar. Equal parts of urine and caustic potash are heated in a test-tube, and, if sugar be present, a deep red brown colour is produced. High-coloured and albuminous urines and those containing phosphates usually darken on boiling with liquor potassæ. If the liquor potassæ contains lead (which it may absorb from the glass it is contained in) urine, when boiled with it, will darken as black lead sulphide is produced, even if no sugar be present.

The last test for sugar which we shall describe is known as the fermentation test. It may also be used to estimate the amount of sugar, and it is in this connection that we shall describe it. It depends on the fact that glucose is changed by fermentation into alcohol and carbonic acid gas. A half-pint bottle is filled half full with urine, and into it is introduced a small piece of dry German yeast. The bottle is then corked, a groove having been first made in the cork to allow of the escape of the carbonic acid gas which is formed. Another bottle of similar capacity is filled with the urine and corked tightly. The two bottles are then placed, side by side, in a warm place and left for 24 hours. At the end of that time the fermentation process is complete. The specific gravity of both samples is now taken, that of the unfermented urine being regarded as the standard. The fermented urine will have been found to have lost some degrees of specific gravity according to the amount of sugar present.

The sugar, which has been acted upon by the yeast, has been converted into alcohol and carbonic acid gas, the latter of which has escaped through the groove in the cork. It has been found that every degree of specific gravity lost represents one grain of sugar in every ounce of urine. To ascertain, then, the amount of sugar passed in 24 hours, we have only to find out the quantity of urine passed in that time. A loss of 15 degrees of specific gravity would give us a total of 1,200 grains of sugar where 80 ounces of urine were passed in the 24 hours ($80 \times 15$).

The nurse need not trouble herself with any other means employed to estimate the quantity of sugar passed.

Before we leave the subject of sugar in the urine it will be well to refer to two substances which may occasionally circulate in the blood in diabetes mellitus and which are believed to be closely connected with the diabetic coma which so often is the cause of death in diabetes. These substances may both be present in the urine and it is important that they should be discovered when they are so present. They are aceto-acetic acid and acetone, the former being decomposed into the latter and carbonic acid gas.

To test for aceto-acetic acid the urine must be fresh and unboiled. To some of the urine in a test-tube add a solution of the perchloride of iron, and if this or its allied acids be present the "ferric chloride reaction" is got, namely, a deep claret-red colour. On boiling the urine this disappears; but where the coloration is due to certain drugs, such as salicylates and carbolic acid, it is not affected by heat. To test for acetone, note first of all the fruity odour usually present in such a case. Hutchison recommends the following test: "To one inch of urine add five drops of a 10 per cent. solution of liquor potassæ. Heat gently. Then drop in a saturated solution of iodine in potassium iodide until the liquid has a yellowish-brown colour. Then add a little more liquor potassæ. Iodoform appears as a yellowish turbidity, which settles down into a crystalline precipitate. It may be recognised by its odour."

To test for blood take an inch of urine in a test-tube and add a drop of tincture of guaiacum and then about half an inch of ozonic ether (which should give off bubbles of gas when poured into the test-tube). If blood is present a blue colour appears at the junction of the fluids. This is caused

### THE EXAMINATION OF THE URINE—*Continued.*

by the guaiac resin being acted upon (oxidised) by oxygen brought to it by the blood pigment which it has taken away from the ozonic ether.

Certain other substances may produce a blue colour when this test is used, but such blue colour appears much more slowly than it does when blood is present, and it appears also simultaneously all through the fluid and not firstly at the junction of the fluids. Pus may give, when present, a greenish blue tint with this test, but it disappears on heating.

To test for the bile pigment pour a little urine on to a plate and, dipping a glass rod into some nitric acid,[1] apply it to the urine, when, at the point of contact, a play of colours is obtained, yellow, violet, blue and green rings being produced. This is known as Gmelin's test.

To test for pus firstly heat the urine. The turbidity does not disappear as it would do if it were due to urates. Take two inches of the pus-containing urine and add one inch of liquor potassæ. A ropy gelatinous mixture is produced. Mucus is distinguished from pus by becoming more fluid when liquor potassæ is added to it.

It will not be necessary for the nurse to test every specimen of urine for blood, bile, or pus, but she should know how to test for these substances when occasion arises. Neither will it come within her province to examine the urine for uric acid and certain rare deposits which require the aid of the microscope for their detection. Indeed, the microscope is a necessity to the proper and complete analysis of the urine; but it stands to reason that it would be quite out of place to refer here to its use in this connection. It may be necessary to estimate the quantity of urea passed in the 24 hours (but it is not likely that the nurse will be asked to do this), and the most convenient apparatus for this purpose is the ureometer of Doremus (also called Southall's) or one of its modifications. The nurse will probably have it pointed out to her in the hospital ward if she wishes to see it. It consists of a bent tube with a long arm which is closed and a short arm which is open and expanded at its free end into a bulb, the whole instrument being conveniently fixed on a wooden stand. The estimation of urea depends on the fact that when urea comes into contact with a solution of hypobromite of soda, a chemical change takes place whereby the urea is split up into water, nitrogen, and carbonic acid. The nitrogen gas is collected in the long arm of the instrument and in about a quarter of an hour it is read off by means of a graduated scale on the long arm, and the appropriate calculation is made.

[1] The nitric acid acts best if it is impure, containing some nitrous acid.

*The following table gives the more important characters of the urine in some of the commoner diseases.*

| | Name of Disease. | Condition of the Urine. |
|---|---|---|
| I. | Gastric catarrh | Quantity normal; high coloured; sp. gr. often raised; acid; urates, oxalates, or phosphates may be deposited. |
| II. | Jaundice | Urine greenish brown in colour; frothy; acid reaction; contains bile; quantity and sp. gr. usually normal. |
| III. | Heart and lung disease | Urine often diminished; dark in colour; acid; high sp. gr.; urates often deposited; albumin often present. |
| IV. | Fevers. general and special | Quantity nearly always diminished; high coloured; usually acid; high sp. gr.; turbid; urates; may be albumin, blood and tube casts; urea increased in amount. |
| V. | Diabetes mellitus | Quantity increased, pale, usually acid, sweet odour; high sp. gr.; sugar in greater or less amount; amount of urea usually increased. |
| VI. | Acute Bright's disease | Quantity diminished or urine may be suppressed; sp gr. at first raised, then lowered; albumin sometimes blood, tube casts, sometimes urates; urea diminished. |
| VII. | Chronic Bright's disease | Urine usually increased in quantity, pale, frothy, sp. gr. low; albumin in small amount or may be absent; no blood; a few tube casts; urea diminished. |
| VIII | Chronic cystitis | Quantity not usually altered, turbid; often alkaline and offensive; large quantities of ropy mucus and pus (muco-pus); often a deposit of phosphates. |
| IX. | Acute gout | Quantity usually diminished; high coloured; strongly acid; urates deposited; may also be uric acid deposited; sp. gr. raised. |

### Hints on How to Start a District Nursing Association.

(*Concluded from page* 44.)

### Other Items Needed by the Nurse.

A district nurse must be provided with a suitable cupboard or chest in which to keep her stock of appliances, dressings, lotions, ointments, etc. The cupboard must be kept locked. A bag is also an absolute necessity for a district nurse, and if she does midwifery she should have a separate bag for these cases. The bag should be light and strong, and should be fitted with a washing lining, which can easily be removed and washed, and with bottles and jars, etc. As to dressings, as far as possible the patients should be expected to find their own dressings, etc., but the nurse must have some at hand in case of emergency and for the very poor. Bandages are made of unbleached calico, by tearing a piece 6 yards long into strips of 2½, 3, 4 inches wide, removing the selvedge. Gifts of white rag, old linen and calico, and even pieces of flannel or flannelette are most useful. Dressings, lotions, and ointments, etc., can be got from most chemists, and the bag and appliances from an instrument maker.

### Appliances for Lending.

A few of the most important things for lending can be got to begin with and the stock increased as required. Bronchitis kettle and stove, bed pans and urinals, ice bag, water bed and water pillow, air cushion, mackintoshes, foot warmer, feeding cups, bed rest.

*Clothing.*—Sheets and blankets, etc. (only lend if absolutely necessary), a couple of flannel night shirts, a couple of flannel night gowns, children's flannel night gowns, baby clothes (lent for one month to destitute cases), flannelette gowns, flannel barries, knitted vests, flannel binders.

### Dressings, etc., for Nurse.

Bag with washing lining, bottles, jars, etc.; Higginson syringe (two if possible), douche tube with syphon bend and glass nozzles, catheter No. 8, soft rubber; clinical thermometer, pan for boiling instruments, white and boracic lint, absorbent wool, sticking-plaster and strapping, oil silk or gutta percha tissue, zinc ointment, boracic ointment, 1 lb.

jars; vaseline, tube of antiseptic lubricant, zinc powder, boracic powder, starch powder, carbolic acid pure, or other disinfectant; methylated spirit, carron oil, olive oil, turpentine, corrosive sublimate tabloids, permanganate of potash.

### Suggested Scale of Fees.

The services of the district nurse are intended chiefly for the poor and working classes subscribing from 2s. to 10s. a year, her services being free of extra charge except for confinements and night duty. Fees for subscribers should be somewhat as follows:—

| | Yearly. | Confinements. | |
|---|---|---|---|
| 1. Labourers, etc. ... | 2s. | 5s. | Visited for 14 days. |
| 2. Artisans ... ... | 3s.–4s. | 7s. 6d. | |
| 3. Farmers ... ... | 5s.–10s. | — | |

Confinements should be booked at least two months in advance and the fees paid. If a doctor is engaged or has to be called in, half fees are charged. Non-subscribers may have the nurse during remainder of current year by paying double the annual subscription; or they may pay so much per week with double fees for confiements:—

| | Per week. | |
|---|---|---|
| 1. Labourers, etc. ... ... | 1s. | One visit charged same as one week. |
| 2. Artisans ... ... ... | 2s. 6d. | |
| 3. Farmers ... ... ... | 5s. | |

The nurse is not expected to sit up at night except in urgent cases, and at confinements (the latter free), but in case of need the following should be the charges:—

| | Per night. |
|---|---|
| 1. Labourers, etc. ... ... ... ... | 4d. |
| 2. Artisans, etc. ... ... ... ... | 6d.–8d. |
| 3. Farmers, etc. ... ... ... ... | 1s.–1s. 6d. |
| Non-subscribers double. | |

It is not a good plan to let the people pay so much for each visit because that limits the nurse's power for doing good. If she feels that each of her visits has to be paid for, she may go less frequently to see a patient than might otherwise be the case if she were at liberty to go as often as she liked. It answers better to have a weekly payment for those who will not become subscribers on the benefit system.

### Form of Annual Report.

When an Association has become fairly established, a report should be printed at the end of each year, an annual meeting held, and copies of the report circulated. The report may be arranged in the following way:—(1) Title—First annual report of the District Nursing Association (2) List of officers—President, Treasurer, Secretary, etc. (3) General report of the nurse's work and anything else of interest. (4) Summary of cases—number of surgical cases, number of medical cases, number of chronic cases, number of maternity cases. (5) Balance sheet. (6) List of subscribers. (7) Rules of Association.

### Early Days.

If the work of the association does not at first seem to make much progress there is no need to be discouraged, for as a rule in most places it takes a little time for the nurse to gain a footing among the people, and she has nearly always to contend with a great deal of prejudice and even shyness. Perhaps the first year there are not as many subscribers among the poor as there ought to be, many of them do not take kindly to the idea of having a stranger to attend to them if ill, and anyhow prefer to wait till they are ill before they part with any money. But once the nurse has successfully nursed a few patients, and has won a few hearts by her skill and kindness, her influence soon spreads. The people no longer look upon her as a stranger, but as a friend to whom they may turn in time of sickness. One neighbour tells another what she did for her and so on, and it very often happens that the very people who were most opposed to her are the first to need her help, and afterwards the most enthusiastic in recommending her to others. Thus the work grows and subscribers increase, especially if there are some energetic and tactful collectors.

### Making Application.

Differences of opinion exist as to whether it is best for the people to go direct to the nurse when applying for her services, or whether they should first obtain an order form from the secretary. As a rule the people will go much more readily to the nurse than they will go to a committee lady; they do not mind telling the former anything, but they shrink from being questioned by anyone else. After all, it is rather a waste of time to have to go to the secretary, who may be busy or out, before they may go to the nurse, and if they produce a Member's card there cannot be much objection to their applying to the nurse direct, especially if the nurse keeps a careful record of her work and reports to the secretary each week. If the plan of applying to the secretary is adopted, exception should be made in the case of urgent calls, when no time must be lost.

### The Nurse's Position.

With regard to the nurse herself, it should be remembered that her position is often a very isolated one, and life in lodgings in a country district is apt to be extremely lonely. She needs friends and a change sometimes, and in many little ways people can do much to help and encourage her. The loan of books and papers is always acceptable, and a few flowers now and then will brighten up her room.

### Specimen Pages of The Nurse's Books.

*Time Book.*

| Date. | Morning. | Afternoon. | Evening. | Night. | Hours. | Visits. |
|---|---|---|---|---|---|---|
| 1st | 9-1.30 | 4.30-7.30 | | | 7½ | 6 |
| 2nd | 9-12.30 | | 6-8 | | 5½ | 7 |
| 31st | | | | | | |
| | | | Total .. .. | | | |
| | | | Daily Average | | | |

*Visit Book.*

| Names of Patients. | 1 | 2 | 3 | 4 | 5 | 6 | 7 | | | | | | | 30 |
|---|---|---|---|---|---|---|---|---|---|---|---|---|---|---|
| Mrs. S. | 11 | 11 | 11 | 1 | 1 | 1 | 1 | X | | | | | | |
| Mrs. B. | 1 | 1 | 1 | 1 | X | | | | | | | | | |
| Mrs. W. | 1 | | 1 | | 1 | O | O | X | | | | | | |
| Mrs. B. | 1 | 1 | 1 | 1 | 1 | 1 | 1 | 1 | 1 | 1 | 1 | 1 | 1 | 1 |
| Mrs. Y. | 1 | | | 1 | | O | | | O | | | | O | X |

*Loan Book.*

| Appliance. | Name and Address. | Lent. | Returned. |
|---|---|---|---|
| Feeding Cup .. | Mrs. Hill, 8 High Street | March 4th .. | April 30th |

### New Books on Nursing.

Home-nursing for Young Housekeepers, with Special Reference to the Care of Infants and Children. By Elizabeth J. Moffett, B.Sc., M.D.Lond., Lecturer to the London School Board and Campden Institute, Kensington, etc. (London: Kegan Paul, Trench, Trübner and Company, Limited. 1903. One vol. 107 pages, 19 illustrations. 1s. 6d.).

Here we have yet another manual on home-nursing, purporting to be for young housekeepers, and combining between the covers of its 107 pages some very useful hints and directions for the successful nursing of cases at home. If this class of book were only more widely and intelligently studied we should hear less of unsuitable and slight cases of illness crowding the out-patient departments of our hospitals and of patients being left without medical assistance until it was too late to be of any service on account of the ignorance of those about them. It is true that most of the good advice given in this little book has been given before, but it is none the less sound for that, and a good thing will bear repetition. The chapter describing the method of isolating infectious cases and disinfecting the room is well and clearly explained. A chapter is devoted to the "common complaints of infants and children," and gives sensible advice as to their treatment, though, perhaps, a few more explanatory words on the quantities of food for various ages up to seven years would have been helpful, so many of these complaints being induced or aggravated by ignorance or carelessness on this point. Carefully divided headings to each chapter make up for the absence of an index, and it would be well if it could find a place on the bookshelf of every wife, mother, and elder sister on whose common sense and promptitude of action so often largely depend the issues of life and death in everyday illnesses and emergencies.

Home Nursing. By Bernard Myers, M.D., C.M. M.R.C.S., L.R.C.P., etc., Lecturer and Surgeon to St. John Ambulance Association. (London: Baillière, Tindall and Cox, 8 Henrietta Street, Covent Garden. 1903. One vol. 131 pages. Fifteen illustrations. 2s. 6d. net.)

Well got up and neatly bound, this unpretending work on home nursing ought to prove very useful to those for whom it is intended. It only aims, as the author says in the preface, at "guiding an untrained nurse in her duties when administering to the sick," and though such things as giving a sponge bath more properly belong to the sphere of a trained nurse, still, no doubt, by carefully following the directions given, any intelligent woman could do it satisfactorily. We must confess, however, that we do not see the necessity for placing "some towels folded lengthwise beneath the mackintosh all round the edges to prevent the water soaking the mattress." With a mackintosh of proper texture and length under the patient, there should be no sort of need to "soak the mattress" at all when washing or sponging a patient; neither do we, even in private houses, as a rule, use a "pail" for this purpose. The directions on p. 20 and p. 21 as to bed-making do not seem quite clear where the sentences occur: "Next comes the under sheet. At times, two or three of these are used together, the top one being removed as may be necessary." And "should the patient tend to kick off the sheet, it should be secured by safety pins at the four corners." Unless the patient happens to be a very young infant, safety pins would be of very little avail, should he "tend to kick," as they are so easily dislodged, and would only result in tearing the sheets and scratching the patient. The book being written for untrained nurses it seems a pity to introduce such words as "hebetude" without an explanation attached to it; and in the otherwise excellent section on "The dressing of wounds," why advise the use of a piece of "antiseptic cotton wool about the size of the fist" to be constantly dipped in the lotion and applied to the wound? Why not much smaller swabs of wool, which having been once used can be thrown away, instead of soiling the lotion again and again? We are glad to note that in the chapter on "Sick-room régime," the amateur nurse is advised to "get someone to relieve her of her charge for an hour or so every day, so that she can have a walk and get some fresh air," and we quite endorse the author's opinion that the "nurse's health also requires attention, as if she breaks down it becomes very awkward." It does indeed! The chapter on elementary human anatomy and physiology, comprising as much information on such an exceedingly wide subject as can be expected in the limited space allotted to it, and the explanatory diagrams, are clear and good. The processes of digestion and the circulation of the blood are well explained, also the "Preparing for a surgical operation at home," an ordeal which, with the aid of this manual, ought to be shorn of half its terrors to the untrained nurse.

Practical Nursing. By Isla Stewart, Matron of St. Bartholomew's Hospital, London, and Herbert E. Cuff, M.D., F.R.C.S., Medical Superintendent North-Eastern Fever Hospital, Tottenham, London. Vol. II. (Edinburgh and London: William Blackwood and Sons. 203 pp., 14 illustrations.) 3s. 6d.

To those who read with pleasure and profit the first volume of this eminently practical work the time has seemed all too long to wait for the issue of the second, and a glance at the names on the title-page is a sure criterion of the excellence of the book. The first nine chapters treat exhaustively of the nursing treatment of medical cases. The section on diphtheria is specially good, and the antitoxin treatment is explained clearly and concisely. Exceedingly lucid, too, are the descriptions of the various forms of aneurism, and the common-sense directions concerning the treatment of phthisis. The chapter on gynæcological nursing, with its minute instructions as to the giving of a vaginal douche, is helpful. Extremely noticeable is the lack of "wordiness" in the whole book. The only chapter in which a few more explanatory words might have been added with advantage seems to be that on enteric fever, when, in describing the outward and visible signs of typhoid during the first, second, and third weeks, the authors do not explain how these symptoms illustrate and coincide with the formation, progress, and healing of the intestinal ulcers, nor mention the characteristic "pea-soup" stools. Furnished with these two volumes a nurse need never be at a loss what to do, from a nursing point of view, for any case that comes under her care. There is no section of her work left untouched, and the best and most up-to-date methods are explained and classified in such a manner as to be at once completely comprehensible. The keynote of all good nursing—the necessity of perfect rest for the patient—is constantly insisted upon in almost every chapter. Careful method is also followed in the arrangement of facts, the opening sentences in each chapter being a brief definition of the disease under consideration; next, the symptoms are elaborated; and then follow the nursing and treatment. Complications and specialities of each disease are indicated by large-typed headings, which catch the eye at once, and in some cases a summary of the most important nursing points is added, so that nothing may be omitted.

### Everybody's Opinion.

**[Correspondence on all subjects is invited, but we cannot in any way be responsible for the opinions expressed by our correspondents. No communication can be entertained if the name and address of the correspondent are not given as a guarantee of good faith, but not necessarily for publication. All correspondents should write on one side of the paper only.]**

### MIDDLE-AGED NURSES.

"A Matron" writes: I think that in the present day when women workers have to take so many responsibilities in the chances and changes of life like men, this foolish, sentimental talk about age limit is absurd. Why cannot women be quiet about such things and remember that it is not the numerical years that count for age with either men or women so much as health and character. Many a woman of 50 is more capable, more active, and much more valuable to her employers than a woman of 30, be she nurse or belonging to any other profession. From an experience of over 20 years in hospital life I would unhesitatingly say that in many ways a nurse of 40 or over is far more valuable than one of 28 or 30 in a house of sickness and trouble; she is also of great help and comfort to a matron, as a sister of wards. Always, of course, providing that she is a good woman and a good nurse.

### THE CLOTHES OF MEDICAL MEN.

"M. R." writes: To all those learned "medicos" who think of taking "Sister's" advice and starting out on their daily round attired in a "white washing suit, with perhaps a white yachting cap," I would say Don't; just imagine such an apparition driving about London, down Piccadilly, or even across the City Road! Why the ghost of Benjamin Binns would not look more startling to nervous people, and whatever would the doctor look like at the end of the day? No doubt there is room for much more care with some medical men in reference to their clothes. They certainly might keep an overall of linen at each infectious case, as the case lasts some time. In my own cases my doctor always kept another coat to wear in the sick-room, which he removed when he washed his hands before putting on his other coat. Of course his top coat and hat were always left in the hall. Then, again, the doctor does not come in such close contact with the patient or bedclothes as the nurse does, and a good doctor would necessarily take every precaution against infection for the sake of others as well as himself.

### NUNS AS NURSES.

"Cecilia" writes: As neither "Trained Nurse" nor myself knows the details of the dispute at Granard Workhouse it seems absurd to try and argue about it, but with your permission I cannot refrain from one final word as to her last letter. She thinks the cases that made the difficulty were midwifery ones and says "no hospital nor workhouse would retain the services of a nurse who declined to perform this part of her nursing duties," meaning the midwifery part. As your correspondent is a trained nurse she should know that there are many trained nurses who absolutely decline to take midwifery cases. If she had instructed as many nurses (fresh from a London hospital with their three years' certificate) as I have over their first midwifery case she would realise what an entirely separate branch of nursing it is and also how some nurses dislike the work. "Trained Nurse" is anxious to show that the nurses who succeeded the nuns at Granard were perfectly free agents "there was no compulsion." But she will not allow any freedom to the nun; she is to be compelled to take midwifery cases whether she will or no, though I contend that the nun has as much right as the nurse to stipulate what kind of work she will do and what she will not do. Again, "Trained Nurse" says "if either nurses or nuns put their own likes before the needs of the patients . . . they fail in their duty." That is true enough if you are talking of individual cases; but between that and stipulating what kind of work you will undertake there is no analogy whatever. Does not every nurse, after her training, consider her own likes and dislikes in deciding what branch of nursing she will follow? After all, gynæcological cases form a very small proportion of workhouse nursing, the majority of the cases are old chronic ones. In Mr. H. Meadows' letter, in the same issue of your paper, referring to his paper on workhouse management, he says, "as the cases they (workhouse nurses) have chiefly to deal with require kindness and care rather than great skill I did lay particular stress on character." Nothing could express better what one feels is the need in workhouse infirmaries, and are not the patients more likely to get kindness and care from the nuns who are there year after year, than from young nurses who leave as soon as possible? The nearest approach to a workhouse nursed by nuns in this country is Nazareth House, Hammersmith, the sisters are always ready to show visitors over it, and those who are accustomed to the ordinary workhouse will see what it is to be looked after by the little sisters of the poor "Trained Nurse," with all her experience, evidently still lacks acquaintance with any of the nursing orders of our Church; if she had the pleasure of knowing some of them she could not remotely hint at a "sanctimonious manner," nor has such ever appeared to me as characteristic of Roman Catholics. She would also find them quite equal to talking professionally even to a trained nurse. In conclusion, the interesting article "An Impression from the Land of the Shamrock," about a nursing home in Dublin in your issue of October 10th, speaks more eloquently than any words of mine can on this subject.

### Presentations.

Kent and Canterbury Nurses' Institute. — An interesting ceremony took place at the Kent and Canterbury Nurses' Institute on Thursday last week, when the past and present nurses of the staff met in order to celebrate the completion of twelve years' work by Miss C. F. Shaw as their lady superintendent, and to present her with a tea-set and case of silver teaspoons as a small token of gratitude for her unfailing kindness to her nurses. During these years the Institute staff has grown from 12 to 46, and various material improvements have been made. The Institute itself has been removed to a larger and more commodious house, which, with the garden attached, is now its own property; a branch nursing home for paying patients has been started at Herne Bay, to which nurses who need change of air are occasionally sent; an affiliation has been arranged with the Royal National Pension Fund for Nurses; and a branch of St. Barnabas Guild has been started, to which many of the Institute and hospital staff belong.

### Where to Go.

Midwives' Institute and Trained Nurses' Club.— Lectures by Dr. Mary Rocke:—October 26th, Infant Feeding; November 2nd, Infants' Diseases, at 3 30 p.m. These lectures are specially for monthly nurses and pupil midwives. Admission 1s. 6d. each, or 2s. 6d. both lectures.

### To Nurses.

**We invite contributions from any of our readers, and shall be glad to pay for "Notes on News from the Nursing World," or for articles describing nursing experiences, or dealing with any nursing question from an original point of view. The minimum payment for contributions is 5s., but we welcome interesting contributions of a column, or a page, in length. It may be added that notices of appointments, entertainments, presentations, and deaths are not paid for, but that we are always glad to receive them. All rejected manuscripts are returned in due course, and all payments for manuscripts used are made as early as possible after the beginning of each quarter.**

### Appointments.

[No charge is made for announcements under this head, and we are always glad to receive, and publish, appointments. But it is essential that in all cases the school of training should be given.]

Cancer Hospital, Fulham Road, London.—Miss Edith M. Shotter has been appointed matron. She was trained at the Sussex County Hospital, where she was afterwards staff nurse, sister, and assistant matron. She has since been matron of the Royal Infirmary, Sheffield.

City Fever Hospital East, Old Swan, Liverpool.—Miss Lilias G. R. Dawson has been appointed deputy matron and Miss Annie Floyd night superintendent. Miss Dawson was trained at Meath Hospital, County Dublin Infirmary, Dublin. She has since been staff sister at the Meath Hospital, Dublin, charge nurse at the City Hospital South, Grafton Street, Liverpool, and ward sister at the City Hospital East, Old Swan, Liverpool. Miss Floyd was trained at Leicester Borough Hospital, and has since been charge nurse at the City Hospital South, Grafton Street, Liverpool.

District Asylum, Westgreen, Dundee.—Miss H. M. Crerar has been appointed matron. She was trained at the Western Infirmary, Glasgow, and has since been sister at the Ayr County Hospital, and assistant matron at the Main Building, County Asylum, Rainhill, Lancashire.

Exeter Sanatorium.—Miss Ella Welsford has been appointed charge nurse. She was trained at Monsall Fever Hospital, Manchester, and has since been charge nurse at the Passmore Edwards Hospital, Acton.

High Wood Ophthalmia Schools.—Miss Emily Baker has been appointed matron. She was trained at the Hospital for Sick Children, Great Ormond Street, London, and has been nurse and matron's assistant at Freeman's Orphanage, Brixton, nurse-matron at Brighton College, and resident nurse at Dean Close School, Cheltenham. She has since been matron of Bridge School, Witham.

Kettering and District General Hospital.—Miss Alice Chapman has been appointed matron. She was trained at Manchester Infirmary, and has since been night superintendent at Bromley Hospital, matron of Rochdale Hospital, and matron of Ashton-under-Lyne District Infirmary.

Lokoja Hospital, North Nigeria.—Miss E. Mitchell has been appointed assistant matron. She was trained at Preston Royal Infirmary. She has since been sister at Stockton-on-Tees Hospital, and at St. Mary's Hospital, Plaistow.

Millfield Seaside Home, Rustington, Sussex.—Miss Grace Kingcott has been appointed matron. She was trained at St. Mary Abbot's Infirmary, Kensington. She has since been assistant matron at the North-Western Hospital, Hampstead, charge nurse at the South-Western Hospital, and recently matron of the City of London House Asylum, Stone.

Ross-on-Wye Cottage Hospital.—Miss Sibyl Dauney has been appointed matron-nurse. She was trained at St. Bartholomew's Hospital, London, and has since done nursing in South Africa as a member of the Army Nursing Service Reserve, and been nurse at Almondsbury Memorial Hospital.

Royal Halifax Infirmary.—Miss Barbara S. Powne has been appointed sister. She was trained at Guy's Hospital, London, and has since been sister of the children's ward at Wigan Infirmary, night superintendent at the Royal Hants County Hospital, Winchester, and sister of the surgical wards at the Royal Victoria Hospital, Belfast.

Royal Hospital for Diseases of the Chest, City Road, London.—Miss Helen F. Cameron has been appointed matron. She was trained at St. Thomas's Hospital, London, and has since been sister at the London Fever Hospital, and matron of the Warrington Hospital.

St. Anne's Home, Herne Bay.—Miss Elizabeth Palmer has been appointed matron. She was trained at Guy's Hospital, London, and has since been housekeeper at the Park Hospital, Hither Green, night superintendent at the Gore Farm Hospital, and acting sister at the Children's Hospital, Highgate.

St. Olave's Union.—Miss Adelaide Bradish has been appointed superintendent nurse. She was trained at St. Olave's Union Infirmary, where she has since been charge nurse. She has also been assistant matron at St. Olave's Union Workhouse.

Soldiers and Sailors Families' Association.—Miss Emily F. C. Eastcott has been appointed "Alexandra" nurse at Devonport. She was trained at the Meath Hospital, Dublin, and the Seamen's Hospital, Greenwich. She has since been staff nurse at Oswestry Accident Hospital, and "Queen's" nurse. She holds the L.O.S. certificate.

Southwark Infirmary, East Dulwich.—Miss May Willis has been appointed night superintendent. She was trained at the London Fever Hospital, Islington, and the Derbyshire Royal Infirmary. She has since been ward sister at the Derbyshire Royal Infirmary.

West Ham and East London Hospital.—Miss Ethel Salmon has been appointed staff nurse. She was trained at North Staffordshire Infirmary and Eye Hospital, Harsthill, Stoke-on-Trent.

### "The Hospital" Convalescent Fund.

The hon. secretary acknowledges, with thanks, the receipt of 4s. 6d. from the "Travel" correspondent of The Hospital.

### Wants and Workers.

The Hospital.—Would the nurse of a cottage hospital care to have The Hospital each week posted Sundays? Address Mrs. Thomas, 1 Nelson Terrace, Victoria Avenue, Wellington, Shropshire.

### TRAVEL NOTES AND QUERIES.

By our Travel Correspondent.

Roads Round Naples (Kathleen).—The roads are very good, only not so ideal as the French. If you remain any length of time it might be worth while to take your cycle, but it would add to the cost considerably and I rather think it might be better to hire in Naples and so spare wear and tear to your own on the journey. I cannot give you the exact cost of the journey by sea till next week, when I shall be in town, but I do not think it would be cheaper on the whole; it is such a long way round and you must be fed all the time.

Bay of St. Malo (Duab).—First-class return, £2 12s. 6d.; second return, £1 19s. The latter is quite comfortable, and in some respects is preferable in the tourist season because it is not so crowded. But by now all crowding will be over. I never think it a good plan to attempt seeing Normandy and Brittany at the same time, when a holiday is limited, but if you are determined to do so your best plan will be to take passage to St. Malo and to return by Havre. The St. Malo crossing is a bad one late in the season, so I should go that way and return by Havre, which is calmer.

### Echoes from the Outside World.

### Movements of Royalty.

THE King, after a day's shooting at Six Mile Bottom, on the estate rented in Cambridgeshire by the Duke of Cambridge, returned to London on Saturday evening, visiting the Lyric Theatre later. On Monday morning his Majesty held a Council at Buckingham Palace, and subsequently received the members of the Alaskan Boundary Tribunal in audience. He heartily congratulated the Commissioners upon the conclusion of their labours. In the afternoon the King left town on a visit to the Marquis of Londonderry at Wynyard Park, and in the evening held a council for the swearing-in of his host as Lord President of the Council. The proprietor of a Devonshire newspaper having called the King's attention to a report to the effect that "his Majesty is a pronounced free-trader, and would regard with great dislike any proposal for taxing the food of the people," his Majesty's private secretary has replied that "the King never expresses any opinion on political matters except on the advice of his responsible Ministers, and that therefore the statement referred to must be inaccurate."

On Sunday the monuments, in white marble, of the late Emperor and Empress Frederick, in front of the Brandenburg Gate, Berlin, were unveiled by the German Emperor, King Edward being represented by Colonel Count Gleichen who handed the Emperor an autograph letter from his Majesty. On the previous evening there was a banquet at the New Palace on the occasion of the confirmation of Princes August Wilhelm and Oscar, to whom the Emperor delivered a remarkable address in which he assured his sons that the angle and turning-point of a human, and especially a responsible and busy life, lies alone in the attitude adopted towards our Lord and Saviour.

The King and Queen of Italy concluded their visit to France on Sunday. On Saturday the Queen made many purchases in Paris, including several hats, a large quantity of lingerie, some bibelots, and a number of toys, including two dressed dolls for the two little Princesses Yolanda and Mafalda. Countess Torinelli asked after the Royal children, and the Queen replied, "I receive news of them four times a day." On the arrival of the King and Queen at Modana the King addressed a telegram to President Loubet conveying the thanks of himself and his consort for the hearty welcome which they had received on the occasion of their visit to Paris.

### The Anglo-French Treaty.

A TREATY of arbitration between England and France has been signed. It provides that questions of a frictional character, or relating to the interpretation of existing troubles which may arise between Great Britain and France shall, if found incapable of settlement by diplomatic means, be referred to the Permanent Court of Arbitration at The Hague. It is stipulated that this arrangement shall apply only to such questions as do not concern the vital interests, or independence, or the honour of the two local contracting parties, and do not affect the interests of third parties. The French press, in commenting on the conclusion of the treaty, which has attracted the attention of the civilised world, attributes it mainly to the visit of King Edward to Paris, and the improved relations between the two countries which have resulted from the event.

### Women's Meeting at Bristol.

ONE of the most interesting gatherings at the Church Congress, which closed on Friday night, was the women's meeting. Colston Hall, which holds 4,000 people, was densely crowded. The Bishop of Bristol, as President of the Congress, was in the chair, and in his introductory speech he said that there was a power, an influence in that hall to make of Bristol the purest, the brightest, the most Christian city on the face of the whole earth; or, on the other hand, to make of it a very hell upon earth. Dr. Browne instanced the influence of women by two personal illustrations. One was that a remark by his mother led him to take holy orders, and the other that a question put to him, which he could not answer, by the lady who afterwards became his wife, made him a student of history. Mrs. Creighton strongly urged her hearers "not to keep themselves to themselves," but to discharge "the duty of neighbours to many who did not live near to them." They had a duty to their town and to their country. If they brought up children well, that was a great service to the country. But women, as well as men, were concerned that the right persons should be chosen to rule the towns, and she entreated them not to neglect their responsibilities in this direction.

### Dr. Elgar's Oratorio.

ESPECIAL interest attached to the Birmingham Festival this year, because of the production of Dr. Elgar's anxiously-awaited oratorio "The Apostles." As the third part—which will deal with the work of the chosen after their Lord's departure—is not yet completed, only the first two were given. These are sub-divided into seven portions. (1) The Calling of the Apostles, (2) by the Wayside, (3) by the Sea of Galilee, (4) the Betrayal, (5) Golgotha, (6) the Sepulchre, and (7) the Ascension. The principal characters are the Saviour, the Blessed Virgin, Mary Magdalene, St. John, St. Peter, and Judas. Dr. Elgar regards Judas as a "misguided zealot," who endeavoured by his action to force Christ to assume that temporal power which he believed his Master possessed, and which it would be beneficial that He should reveal. Many of those who listened to the performance last week are of opinion that they were present at the birth of a masterpiece, though naturally such a stupendous work requires deeper study than is possible at a single hearing. Several passages haunt the memory, notably Christ's delivery of the Beatitudes, interrupted by comments from the disciples and the multitude; the heartbreaking prayer of Mary Magdalene; and the finale of the first part, "Turn you to the stronghold, ye prisoners of hope." The performance was a fine one, and the respective performers gave reverent and beautiful readings of their parts, and the choir and orchestra were respectively deserving of high praise.

### Society of Oil Painters.

THE autumn Exhibition of the Society of Oil Painters, in Piccadilly, which was opened on Monday, has a few large and important pictures of merit and several small ones equally deserving. There are, perhaps, more canvases than usual this season which might have been excluded with benefit to the gallery, and a great many which follow so closely on the beaten track that almost their exact counterparts have been seen for years. Sir James Linton has an impressive, if somewhat theatrical, "Casket Scene"; Sir George Reid a poetic reproduction of the oft-painted town of "Durham"; Mr. Lavery, "Summer," a girl and a child overlooking a large expanse of sea and receding chalk cliffs. Neither Mr. Fulleylove's "Jerusalem" nor Mr. Aumonier's "Cambridgeshire Farm" should be passed by. Amongst less noticeable pictures are the "Cloudless Horizon," by Mr. Garrido—a happy child presumably painted just before breakfast; Mr. Douglas Almond's "Waiting for an Audience," a man in a chair formally placed against a wall just by a closed door; and Mr. Edgar Bundy's "Fishwife." There are a few clever works which advertisement managers in search of new and taking pictures to "push" their goods ought not to miss.

### For Reading to the Sick.

"THERE IS A BLESSED HOME."

There is a blessed home
Beyond this land of woe,
Where trials never come,
Nor tears of sorrow flow;
Where faith is lost in sight,
And patient hope is crown'd,
And everlasting light
Its glory throws around.

There is a land of peace,
Good Angels know it well;
Glad songs that never cease
Within its portals swell;
Around its glorious Throne
Ten thousand Saints adore
Christ, with the Father One
And Spirit, evermore.

*S. H. Baker.*

There is in every human heart a craving for happiness. Men seek it by day and by night, yet never are satisfied, for all earthly happiness is limited both in measure and in duration. It is strange that though the human heart is a vessel very finite in its dimensions, nothing short of infinite happiness can fill it. God knows this and God has willed it so. "The well is deep." "We are made for Thee, O God, and our hearts are restless until they find Thee." "What have I in heaven, and beside Thee what do I desire upon earth? Thou art the God of my heart, and my portion for ever."

We are apt not to think sufficiently often of the happiness of heaven. To do so is most beneficial to us, for it fills us with love for God and gives us courage to work and patience to face the trials and pains of life. To think of hereafter is an especial consolation to the sick. "Lift up your eyes to heaven," "to the city of God, of which glorious things are spoken." There is your home: there a place is ready for you. The essential happiness of heaven is the vision of God, face to face, for ever. "We shall see Him as He is." Aided by the light of glory, without which "no one can see God and live," we shall for ever contemplate his goodness, and wisdom, and the harmony of His infinite perfections, while by that very contemplation a song of praise, "Holy, holy, holy," is drawn forth.

Think again that in heaven there is an absence of all pain. "The former things are passed away," "the voice of weeping is not heard," for "God shall wipe away all tears from their eyes." All this is much and splendid, but it is only a glimpse. "Eye hath not seen, nor ear heard, neither hath it entered into the heart of man to conceive the things that God hath prepared for them that love Him." *Anon.*

Thou hast no shore, fair ocean!
Thou hast no time, bright day!
Dear fountain of refreshment
To pilgrims far away!
Upon the Rock of ages
They raise thy holy tower;
Thine is the victor's laurel,
And thine the golden dower.

O sweet and blessed country,
The home of God's elect!
O sweet and blessed country
That eager hearts expect!
Jesu, in mercy bring us
To that dear land of rest;
Who art, with God the Father
And Spirit, ever blest. Amen.

*J. M. Neale, D.D.*

### Notes and Queries.

FOR REGULATIONS SEE PAGE 21.

*Agreement.*

(33) Having joined a "Private Nurses' Home," after nursing for about two months for the Home, I was asked to sign a paper to certify "that I would not nurse on my own account, after leaving the Home, within a radius of 10 miles." This paper bears only a penny stamp, and I should feel obliged if you could inform me if this is legal.—*Anxious.*

The question of stamp does not arise. If you signed a letter, agreeing not to nurse on your own account within a radius of 10 miles after leaving the Home, you are bound by it.

*Children's Nurse.*

(34) Will you kindly tell me if (1) there are any other institutions, excepting the Norland Institute, where children's nurses are trained? (2) Is there any home where an imbecile girl of 16 could be received? Her parents, who are working people, could afford to pay a little for her. (3) Could you tell me when the Midwives Act comes into force? How it will affect untrained women now in practice, and where they may be registered if it is necessary, and if there is any charge?—*M. M. B.*

(1) Yes, the Liverpool Ladies' Sanitary Association, 8 Sandon Terrace, Liverpool; Sesame House for Training Kindergarten Mistresses and Lady Nurses, 43 Acacia Road, St. John's Wood; and the Convalescent and Permanent Home, Uplands, Loughton, Essex. (2) Apply to the Medical Superintendent, Darenth Schools for Imbecile Children, Dartford, Kent. (3) The Midwives Act is already in force, but more stringent regulations will be observed after April, 1905. Apply to the Secretary, the Central Midwives Board, 6 Suffolk Street, Pall Mall, S.W., for full particulars.

*Work.*

(35) Will anyone tell me how I can obtain a post as worker in a convalescent home? I have had institutional experience, but no hospital training.—*Sister.*

You had better advertise.

*Swedish Massage.*

(36) Can I learn massage in Sweden? What would be the cost? Also is there a Swedish training school in London?—*A. A. C.*

Can you give me the address of a good college in Stockholm where a short course of medical gymnastics could be obtained?—*M. B.*

Apply to the Secretary, the Royal Central Gymnastic Institution, Stockholm. The course of instruction is free to some students.

*Electro-Therapeutics.*

(37) I shall be grateful if you will tell me where I can get a few lessons in electro-therapeutics. I am having a two years' course, practical and theoretical, in massage and medical movements, but we do not learn to use electrical instruments.—*A Gymnast.*

The Secretary, the Incorporated Society of trained Masseuses, 12 Buckingham Street, Strand, W.C., might be able to recommend a private teacher.

*Maternity Fees.*

(38) Will you kindly tell me if I can claim my fee? A lady engaged me for a month, but the confinement came several months before it was expected, and I was unable to go to her.—*M. A. H.*

Usually a nurse receives half fees in such cases.

I was engaged to attend a lady in October. Her husband, a solicitor, has just written and informed me that my services will not be required, as his wife has had a miscarriage. Will you kindly tell me what fee is usually claimed in such cases?—*A. J.*

See reply to *M. A. H.*

**Standard Nursing Manuals.**

"The Nursing Profession: How and Where to Train." 2s. net; 2s. 4d. post free.

"Nursing: Its Theory and Practice." (Revised Edition). 3s. 6d. post free.

"Elementary Anatomy and Surgery for Nurses." By William McAdam Eccles, M.D.Lond., M.B. 2s. 6d. post free.

"Elementary Physiology for Nurses." By C. F. Marshall, M.D., B.Sc., F.R.C.S. 2s. post free.

"Ophthalmic Nursing." By Sydney Stephenson, M.B., F.R.C.S., 3s. 6d. net; 3s. 10d. post free.

"Nursing in Diseases of the Throat, Nose, and Ear." By P. Macleod Yearsley, F.R.C.S.Eng., M.R.C.S. 2s. 6d. post free.

"Fevers and Infectious Diseases." 1s. post free.

